# Deiodinases in thyroid tumorigenesis

**DOI:** 10.1530/ERC-23-0015

**Published:** 2023-04-13

**Authors:** Maria Angela De Stefano, Tommaso Porcelli, Martin Schlumberger, Domenico Salvatore

**Affiliations:** 1 Department of Public Health, University of Naples “Federico II”, Naples, Italy; 2 Department of Endocrine Oncology, Gustave Roussy and University Paris-Saclay, Villejuif, France; 3 CEINGE Biotecnologie Avanzate Scarl, Naples, Italy

**Keywords:** deiodinases, thyroid hormones, follicular thyroid cancer, papillary thyroid cancer, anaplastic thyroid cancer

## Abstract

The three deiodinase selenoenzymes are key regulators of intracellular thyroid hormone (TH) levels. The two TH-activating deiodinases (type 1 deiodinase and type 2 deiodinase (D2)) are normally expressed in follicular thyroid cells and contribute to overall TH production. During thyroid tumorigenesis, the deiodinase expression profile changes to customize intracellular TH levels to different requirements of cancer cells. Differentiated thyroid cancers overexpress the TH-inactivating type 3 deiodinase (D3), likely to reduce the TH signaling within the tumor. Strikingly, recent evidence suggests that during the late stage of thyroid tumorigenesis, D2 expression raises and this, together with a reduction in D3 expression levels, increases TH intracellular signaling in dedifferentiated thyroid cancers. These findings call into question the different functions of TH in the various stages of thyroid cancers.

## Introduction

**Figure 1:**
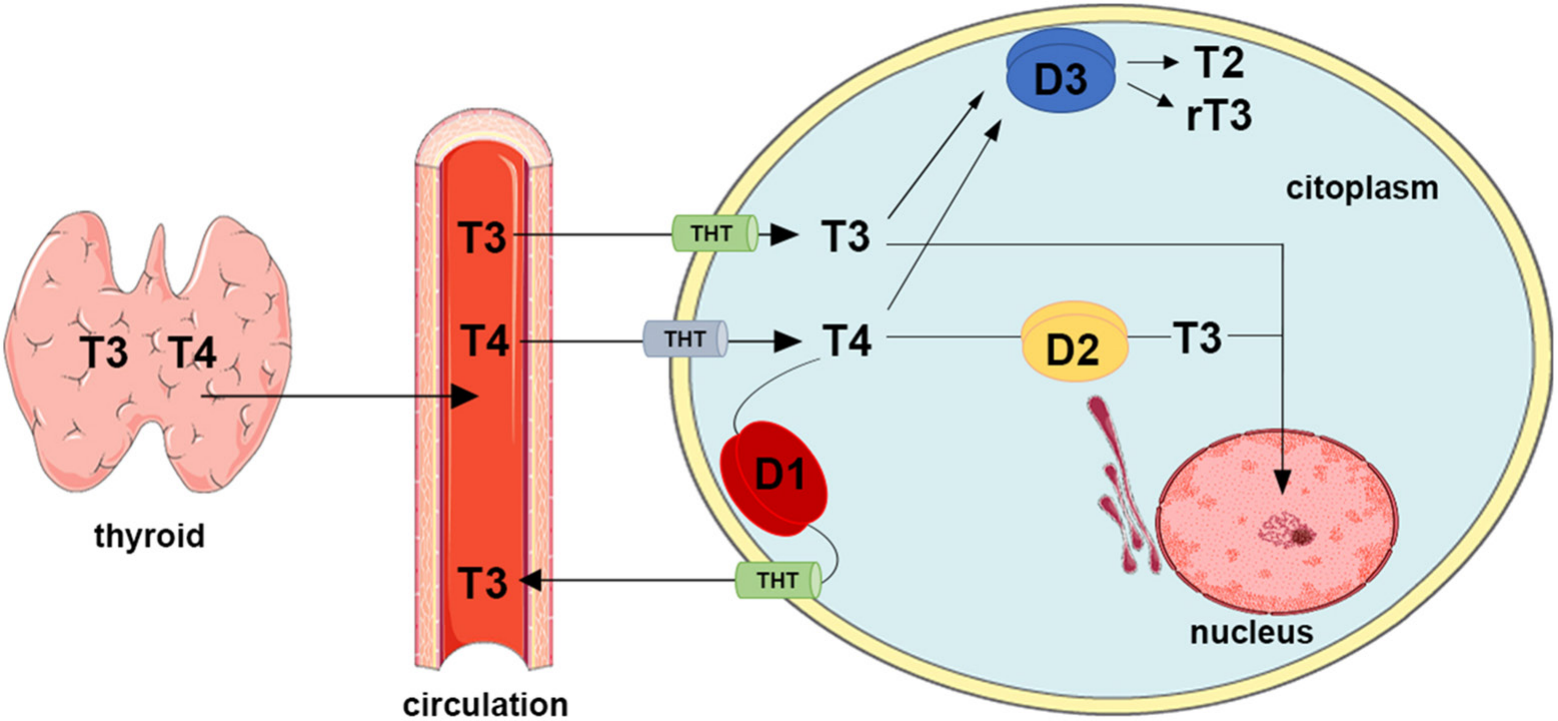
Schematic design of deiodinase expression and function in the cell. The thyroid gland secretes T4 and T3 into circulation. Thyroid hormones enter the cell through thyroid hormone transporters and are subsequently metabolized based on the cell-specific deiodinase expression. D1 is located at the cell membrane and activates T4 into T3, but almost the total amount of D1-derived T3 is released in the systemic circulation. Conversely, D2 acts within the endoplasmic reticulum, close to the cell nucleus, and operates to increase the nuclear T3 concentrations on top of the serum-derived fraction of T3. D3 is also located at the cell membrane but inactivates both T4 into rT3 and T3 into T2, thus lowering the T3 nuclear concentration. D1, type 1 deiodinase; D2, type 2 deiodinase; D3, type 3 deiodinase; THT, thyroid hormone transporter.

Deiodinases are three thioredoxin fold-containing selenoenzymes essential for homeostasis and control of both circulating and intracellular thyroid hormone (TH) levels. Type 1 (D1) and type 2 (D2) deiodinases convert the prohormone T4 into the active form T3 through outer ring deiodination. Their activity accounts for about 80% of the total daily T3 production in humans, while the remaining 20% of T3 and the total daily amount of T4 are produced by the thyroid gland. Type 3 deiodinase (D3), conversely, inactivates both T4 to reverse T3 (rT3), and T3 to T2 through inner ring deiodination (Bianco *et al.* 2002, Callebaut *et al.* 2003). D1 is located in the plasma membrane, whereas D2 is located in the endoplasmic reticulum, and their different subcellular location reflects their different function. In fact, the D1-derived T3 is thought to exit the cells to supply the extracellular compartment, while most of the D2-derived T3 remains in the intracellular milieu and has easier access to the nucleus to bind the T3 nuclear receptors. D3 is located in the plasma membrane and is responsible for the clearance of two-thirds of body T3 by inactivating it at both intra- and extracellular levels (Bianco *et al.* 2002) (Fig. 1).

According to their specific role in TH homeostasis, deiodinases are differently expressed among human tissues. D1 is stably found in the thyroid, liver, and kidney. Conversely, D2 and D3 are found in a larger number of tissues, in many of which their expression levels change in a temporal-specific fashion to customize intracellular TH concentration in response to various stimuli, such as metabolic stress (Simonides *et al.* 2008, Ferdous *et al.* 2020), diseases (Faber *et al.* 1983, Mebis *et al.* 2007), injuries (Mancino *et al.* 2020, De Stefano *et al.* 2021), and normal physiological cell processes (Mancino *et al.* 2020). In such contexts, deiodinases provide finely tuned intracellular TH concentration, irrespectively from the stable circulating TH levels guaranteed by the hypothalamus–pituitary–thyroid axis. As an example, skeletal muscle finely modulates D2 and D3 levels to achieve cell-specific TH concentration during development, homeostasis, and regeneration processes. D3 is highly expressed in the early phase of myogenesis and is required for myoblasts proliferation, while D2 is expressed in the late phase of myogenesis and is required for the induction of differentiation muscle genes (Dentice *et al.* 2014, Ambrosio *et al.* 2017).

Modulation of deiodinase expression also occurs in several cancers vs their normal corresponding tissues (Nappi *et al.* 2021). Increased expression of D3 was found in colon cancer (Dentice *et al.* 2012), basal cell carcinoma (Di Girolamo *et al.* 2016, Miro *et al.* 2017), hemangiomas, gastrointestinal stromal tumors (GISTs), glioblastomas, and ovarian cancer among others (Moskovich *et al.* 2021) and in many human cancer-derived cell lines (Curcio *et al.* 2001, da-Silva *et al.* 2007). In these contexts, D3 upregulation might be driven by different oncogenic pathways, such as WNT-β-catenin, Sonic hedgehog, and transforming growth factor-beta (TGF-β) (Nappi *et al.* 2021). In D3-overexpressing tumors, the maintenance of low intracellular TH levels is necessary for tumor growth. In fact, in both cellular and mouse models, the depletion of D3 led to a reduction in cancer proliferation, and treatment with T3 was found to inhibit tumor growth both *in vitro* and *in vivo* (Dentice *et al.* 2012, Di Girolamo *et al.* 2016, Moskovich *et al.* 2021). However, several pieces of evidence suggest that, in some cancers, late-stage progression may require an opposite (i.e. increased) intracellular TH concentration compared to their early-stage phases. This switch from low to high intracellular T3 levels is obtained by the downregulation of D3 (first observed in colon cancer progression (Dentice *et al.* 2012), and a concomitant upregulation of D2 (formally demonstrated in skin tumorigenesis (Miro *et al.* 2019). We found that late-stage squamous cell cancer (SCC) increases D2 expression to increase intracellular T3 levels: T3 treatment increased the invasiveness of advanced SCC while, in the opposite direction, D2 depletion attenuated the metastases formation, revealing that D2-derived T3 is needed for SCC progression (Miro *et al.* 2019, Nappi *et al.* 2020).

In this review, we focus on the expression of deiodinases in thyroid neoplasias and on how their expression varies between benign lesions, differentiated cancers, and ultimately undifferentiated cancers. Deiodinase mRNA expression was the more frequently studied parameter, but we reported also activity whenever it was measured. We also discuss, in relation with data from an accompanying paper (De Stefano *et al.*), the molecular mechanisms that may alter the expression of deiodinases during late thyroid tumorigenesis and how changes in intracellular T3 levels may impact on the behavior of thyroid cancer cells.

## Deiodinase expression in normal and hyperfunctioning thyroid tissues

Thyroid cell expresses under normal conditions both D1 and D2, but not the D3 enzyme (Galton *et al.* 2021). While it was long known that the thyroid expresses D1 (Berry *et al.* 1991, Nishikawa *et al.* 1998), the expression of D2 in thyroid tissue was unknown until 1996 when we discovered that *DIO2* mRNA levels were 50- to 150-fold higher in human thyroid than in human placenta (Salvatore *et al.* 1996). The presence of D2 in the human thyroid gland was previously unknown because all the assays for D1 and D2 enzymatic activities were conducted – up to then – on rodent samples or in rat-derived cell lines (i.e. FRTL-5). It then appeared that the rodent *Dio2* promoter lacks binding sites for the thyroid transcription factor-1 (TTF-1), a homeodomain protein essential for human thyroid organogenesis and maintenance of thyroid cell specificity. Unlike the rodent *Dio2*, the human *DIO2* 5′-flanking region hosts two critical DNA binding sites for TTF-1, that account for the much higher D2 expression in the human thyroid vs mouse tissue (Gereben *et al.* 2001).

Both D1 and D2 expression significantly increase compared to normal thyroid tissue in Graves’ disease and hyperfunctioning/toxic adenomas, two thyroid hyperproliferative diseases in which the thyroid-stimulating hormone (TSH)-receptor pathway is highly active (Salvatore *et al.* 1996, Laurberg *et al.* 2007, Ito *et al.* 2011). However, their upregulation is thought to follow two major different mechanisms. D1 is strongly upregulated by T3 at the transcriptional level through the binding of the T3 receptor to two distinct thyroid hormone response elements in the *DIO1* gene promoter region (Toyoda *et al.* 1995). Differently, the increase in D2 expression mainly depends on the activation of the TSH-receptor pathway, which activates the synthesis of cyclic adenosine monophosphate (cAMP) (Field *et al.* 1987). The upregulation of D2 depends in turn on cAMP that binds to a cAMP response element in the *DIO2* promoter region (Silva & Larsen 1983, Bartha *et al.* 2000). Notably, the TSH-dependent cAMP pathway also increases the mitotic activity of thyroid cells, causing goiter in Graves’ disease and cell growth of the toxic adenomas, and is paralleled by the induction of other thyroid-specific proteins such as sodium/iodide symporter and thyroglobulin (Dumont *et al.* 1992, Toyoda *et al.* 1995). Therefore, in thyroid cells that proliferate in response to TSH stimulation, D2 upregulation is corresponding to what occurs for other thyroid-specific differentiation markers.

What is the role of deiodinases in normal thyroid cells? D1 is constitutively expressed in normal thyroid follicular cells and contributes to thyroidal T3 production, as well as iodide recycling by deiodination of rT3 and sulfated iodothyronines (Maia *et al.* 2011). The D2 activity is closely related to TSH stimulation and is likely to provide a supplemental amount of T3 to the total thyroidal secretion in cooperation with D1 (Salvatore *et al.* 1996). Whether deiodinases-produced T3 contributes to the T3-bound thyroid receptor fraction in thyroid cells is presently unknown. The overall contribution to the T3 secretion by D1 and D2 is reflected by the decrease in the T4/ T3 molar ratio from 15:1 in thyroglobulin-bound hormones to 11:1 in the thyroid output, which is observed in Graves’ disease and hyperfunctioning thyroid nodules (Salvatore *et al.* 2022).

## Non-functioning follicular thyroid adenomas and carcinomas have a similar deiodinase expression profile

Polyclonal hyperplasia is commonly found within nontoxic multinodular goiters (MNG) and is the result of the non-clonal proliferation of multiple cells (Kopp *et al.* 1994). Analyses of deiodinase expression in MNG of patients with normal serum TSH levels indicate that D1 and D2 show similar expression and activity in MNG when compared to normal thyroid tissue (Salvatore *et al.* 1996, Pereira *et al.* 2012). Conversely, follicular thyroid adenomas (FTA) are monoclonal and arise from the proliferation of a single cell after the acquisition of a growth advantage due to the accumulation of genetic mutations (Kondo *et al.* 2006). Non-functioning FTA may lose the ability to incorporate iodide, in which case they appear as ‘cold nodules’ at thyroid scintigraphy, and this is related to a decreased expression of sodium iodine symporter (NIS). In such non-functioning FTA, the expression of the other thyroid functional proteins such as thyroid peroxidase (TPO), thyroglobulin, and TSH receptor is maintained (Russo *et al.* 2001). Few studies of small-sized groups of non-functioning FTA showed that both D1 and D2 gene expression and activity are similar (Arnaldi *et al.* 2005) or increased (de Souza Meyer *et al.* 2005, Pereira *et al.* 2012) when compared to normal thyroid tissue, with similar TSH levels.

Both FTA and follicular thyroid cancer (FTC) are frequently driven by point mutations in *RAS* genes or *PPARG* fusion genes and share common cytological features and histological architecture (Marques *et al.* 2002, Nikiforova *et al.* 2003, Kondo *et al.* 2006). However, FTCs are invasive lesions that invade the tumor capsule, the thyroid gland capsule, and/or tumor vessels (D'Avanzo *et al.* 2004). Compared to normal thyroid tissue, FTC may show various degrees of D1 expression and similar levels of D2 expression (Arnaldi *et al.* 2005, de Souza Meyer *et al.* 2005). This is consistent with the low MAPK-signaling associated with *RAS* and *RAS*-like mutations and with the maintained expression of other markers of thyroid differentiation. Initial studies of D1 in human specimens of FTC reported normal to lower activity and expression of the 27-kDa substrate-binding subunit of D1, which was stimulated in two FTC cell lines by retinoic acid (Köhrle *et al.* 1993, Schreck *et al.* 1994). In a subsequent series of 11 FTA and 15 FTC, there were no significant differences in the expression level of D1 and D2 compared to surrounding normal thyroid tissue in any of these tumors (Arnaldi *et al.* 2005). Vice versa, D1 expression was significantly upregulated in a smaller series of seven FTA and six FTC compared to healthy thyroid tissue (de Souza Meyer *et al.* 2005). Increased *DIO1* mRNA levels were paralleled by increased D1 activity in FTA and in samples of FTC metastases. This study also reported a D2 expression in FTC similar to normal thyroid tissue, but D2 activity was consistently higher in FTC than in surrounding tissue.

The presence of T4-to-T3 converting deiodinases in FTC was also described in patients with large tumor masses in which the amount of D1 and/ or D2 was so elevated (due to the elevated tumor burden) that it led to decreased serum T4/T3 ratio. Higher expression levels of D1 and D2 than in normal thyroid tissue were found in at least four FTC from patients with increased serum T3 levels in three different studies (Kim *et al.* 2003, Takano *et al.* 2006, Miyauchi *et al.* 2008). A general observation in patients with hyperfunctioning FTC is a reduction in serum T4 and an increase in serum T3 concentrations that depend on an increased T4-to-T3 conversion. In most of these hyperfunctioning FTC, an activating mutation of the TSH receptor gene or of the Gsα gene was found (Brunetti *et al.* 2000).

## Papillary thyroid carcinoma expresses D3 and downregulates D1 and D2

Papillary thyroid cancers (PTCs) are driven in about 50–60% of cases by the *BRAF^V600E^* mutation, while 5–20% of cases show fusion genes such as *RET/PTC*, *PAX8-PPARG,* and *NTRK1/3* rearrangements, and 10–15% show *RAS* mutations (Cancer Genome Atlas Research 2014, Fagin & Wells 2016). These oncogenic driver mutations are mutually exclusive and lead to significantly different signaling consequences. PTC harboring *RAS* and *RAS*-like mutations (such as *PAX8-PPARG* and *BRAF^K601E^*) show follicular architecture and are defined as follicular variant-PTC (FV-PTC). They retain most of the functional properties of normal thyroid cells and have a deiodinase expression profile similar to FTA and FTC (Arnaldi *et al.* 2005). Differently, PTC driven by *BRAF^V600E^* and *BRAF^V600E^*-like mutations (such as *RET/PTC* and *BRAF* fusion genes) show a higher MAPK-signaling activity compared to *RAS*-driven tumors. As a consequence, they lack expression of thyroid differentiation genes that include those involved in iodide uptake and metabolism, and the thyroid deiodinases D1 and D2 (Cancer Genome Atlas Research 2014).

The first observation that D2 expression and activity are decreased in six PTCs came from a study by Murakami *et al.* (2001). In a subsequent analysis, Ambroziak *et al.* (2005) found a robust decrease in *DIO2* mRNA in 8 out of 10 PTC samples and a concomitant downregulation of *DIO1* mRNA levels. Similarly, Arnaldi *et al.* (2005) reported a significant downregulation in both *DIO1* and *DIO2* in a series of 26 PTC compared to normal thyroid tissue. Further insights came from a series of 14 PTC (de Souza Meyer *et al.* 2005) that showed a strong reduction of *DIO1* expression in all PTC samples compared to healthy thyroid tissue and a reduction in D1 enzymatic activity that significantly correlated with the *DIO1* mRNA reduction. The decline in both the T3-producing D1 and D2 in PTC can be simply the results from a reduction of D1 and D2 expression related to oncogenic transformation. However, two studies found that human PTCs significantly increase their D3 expression levels (Romitti *et al.* 2012, Romitti *et al.* 2016) and activity (Romitti *et al.* 2016), suggesting that PTC tumorigenesis may benefit from low intracellular TH levels. In a study of 26 PTC samples (Romitti *et al.* 2012), the D3 expression levels were increased compared to adjacent normal tissue – in which D3 was normally absent (Table 1). Furthermore, the *BRAF^V600E^*-mutated PTC samples showed significantly higher D3 expression and activity than BRAF-wild type controls.

**Table 1:** Summary of deiodinase changes in human thyroid malignancies.

Type of cancer	D1	D2	D3	Author/year
Follicular thyroid cancer	N/A	Increased	N/A	Kim *et al.* (2003)
Follicular thyroid cancer	Increased	Increased	N/A	Arnaldi *et al.* (2005)
Follicular thyroid cancer	Increased	Increased	N/A	de Souza Meyer *et al.* (2005)
Follicular thyroid cancer	Increased	Increased	N/A	Takano *et al.* (2006)
Follicular thyroid cancer	Increased	Increased	N/A	Miyauchi *et al.* (2008)
Papillary thyroid cancer	N/A	Decreased	N/A	Murakami *et al.* (2001)
Papillary thyroid cancer	Decreased	Decreased	N/A	Arnaldi *et al.* (2005)
Papillary thyroid cancer	Decreased	Decreased	N/A	Ambroziak *et al.* (2005)
Papillary thyroid cancer	Decreased	N/A	N/A	de Souza Meyer *et al.* (2005)
Papillary thyroid cancer	N/A	N/A	Increased	Romitti *et al.* (2012, 2016)
Anaplastic thyroid cancer	Decreased	Increased	Decreased	De Stefano *et al*., accompanying manuscript

The deiodinase expression profile was also studied in cell line models of PTCs, and a similar profile was observed when compared to the corresponding human tumors. Indeed, *DIO1* and *DIO2* were found significantly downregulated in the PTC cell line NPA-87 compared to normal thyroid cells (Arnaldi *et al.* 2005). Moreover, the D3 expression and activity were significantly increased in a PTC cell line harboring the *BRAF^V600E^* mutation (K1 cells), compared to normal thyroid cells. In K1 cells, reduction in *DIO*3 mRNA by RNA interference caused a reduction in cyclin D1 levels, a cyclin involved in the control of the cell cycle (Romitti *et al.* 2016). The molecular mechanisms behind D3 induction in PTC are still unknown. However, it has been demonstrated that mutated *BRAF^V600E^* increases the *TGFB1* expression (Riesco-Eizaguirre *et al.* 2009, Knauf *et al.* 2011) and that in different cell contexts, TGF-β induces the transcription of human D3 through a Smad-dependent pathway (Azouzi *et al.* 2017).

Overall, these data support the idea that a low intracellular TH signaling is functional for PTC growth or maintenance. The low TH signaling is achieved not only by abolishing intra-thyroidal T3 production (low D1 and D2 activities) but also by a pro-active tool which consists in D3 activation, thus preserving PTC cells from the TH entry from blood circulation. The molecular signature controlled by T3 in PTC cells is still unknown.

## Anaplastic thyroid cancer expresses D2

**Figure 2:**
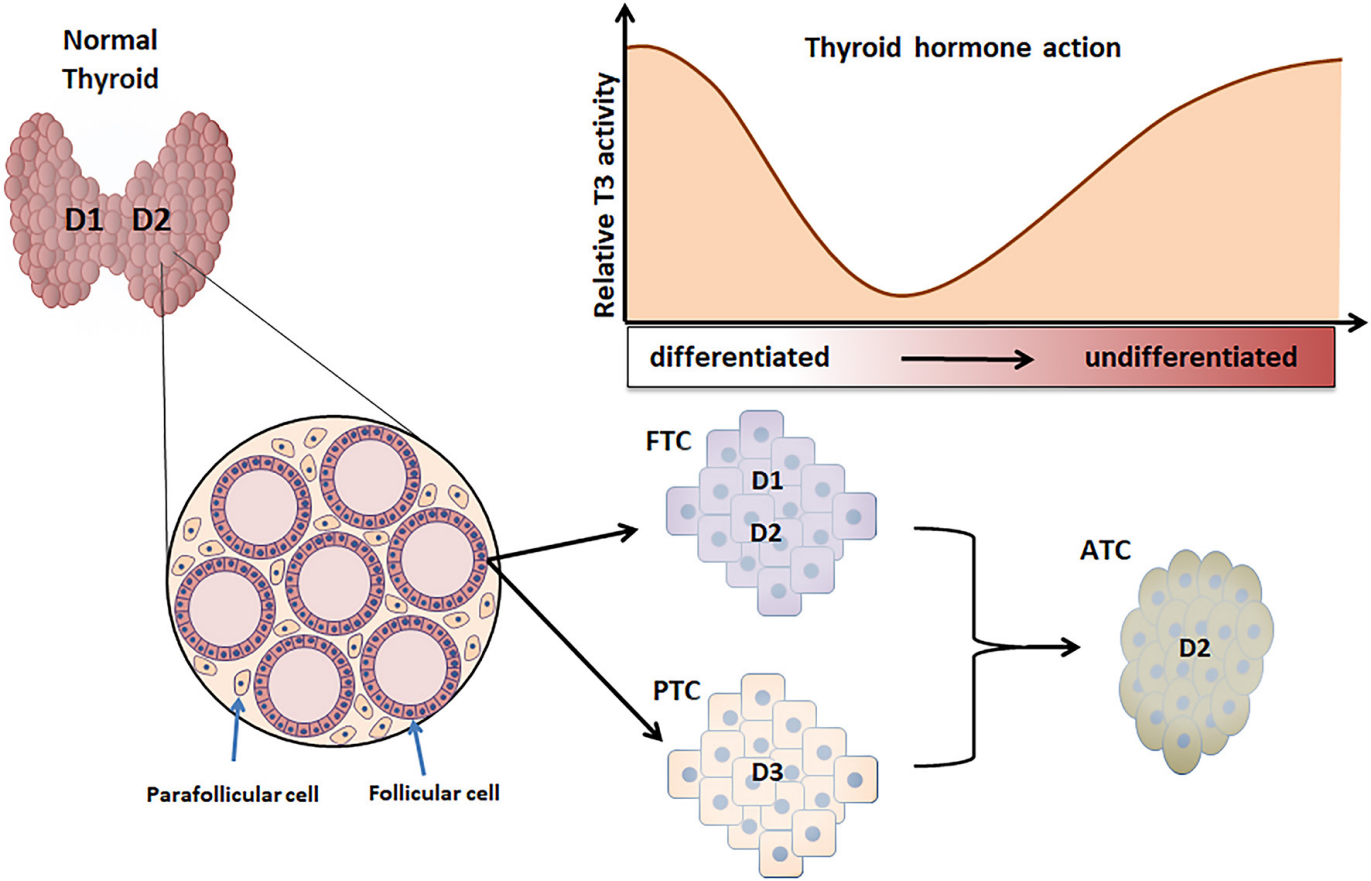
Schematic representation of deiodinase expression and thyroid hormone levels in thyroid tumorigenesis. The diagram shows the deiodinase expression and their variations along the major steps of thyroid tumorigenesis. ATC, anaplastic thyroid cancer; D1, type 1 deiodinase; D2, type 2 deiodinase; D3, type 3 deiodinase; FTC, follicular thyroid cancer; PTC, papillary thyroid cancer.

Anaplastic thyroid cancer (ATC) represents the end result of the dedifferentiation of follicular cell-derived thyroid cancers (Fagin & Wells 2016) (Fig. 2). This is a rare cancer (<1% of all thyroid cancers), but it is an aggressive and rapidly progressive tumor that accounts for one-third of all deaths from thyroid cancer (Landa *et al.* 2016). The accumulation of a high burden of mutations plays a key role in the progression of differentiated thyroid cancer to the undifferentiated state and determines the loss of thyroid-specific proteins, such as thyroglobulin, TPO, and TSH-receptor, and the two thyroid-specific transcription factors TTF-1 and PAX-8 (Fabbro *et al.* 1994, Nikiforov 2004). Initial characterization of deiodinase expression in ATC showed that D1 was undetectable in both human ATC samples and cell models (namely, HTh 74 and C 643) (Schreck *et al.* 1994), a finding that is in agreement with D1 as a thyroid differentiation marker. However, a subsequent study of both D1 and D2 activity in a single sample of human ATC showed a significant increase of T4-to-T3 conversion in cancer tissue as compared to surrounding normal thyroid tissue for both enzymatic assays (de Souza Meyer *et al.* 2005). In addition, immunohistochemistry in one human ATC sample showed no D3 staining (Romitti *et al.* 2012) suggesting a change in the deiodinase expression profile as compared to PTC.

**Figure 3:**
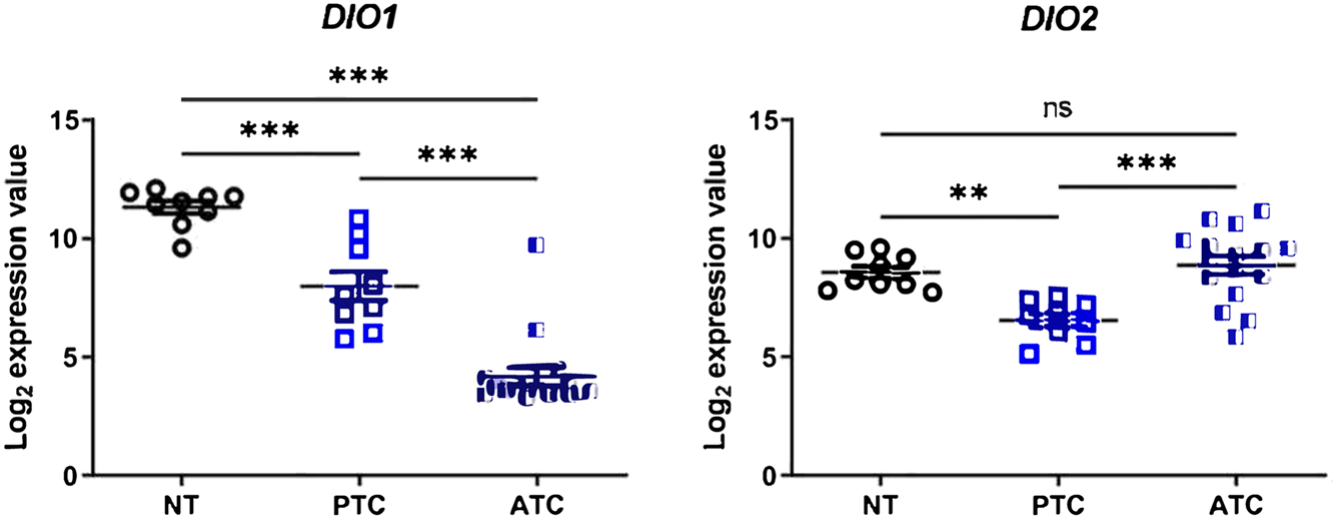
Array analysis of type 1 deiodinase gene (*DIO1*) and type 2 deiodinase gene (*DIO2*) expression in human thyroid cancer from the series GSE3467 and GSE76039 (Landa *et al.* 2016) analyzed using Expression Console. *P < 0.05; **P < 0.01; ***P < 0.001.

New insights on deiodinase expression in ATC came from data derived from two studies on ATC tumorigenesis. In a series of 20 human ATC (Landa *et al.* 2016), the 16-gene thyroid differentiation score derived from the TCGA study (Cancer Genome Atlas Research 2014) (that includes D1 and D2 and reflects the differentiation state of a given thyroid tumor) was used to analyze the effect of the ATC driver mutations on thyroid cancer cell metabolism and specification compared to a group of 9 PTCs. Through the analysis of the published dataset, we observed much lower levels of D1 mRNA in ATC compared to PTC but, surprisingly, increased levels of D2 expression, that were similar to levels found in normal thyroid tissue (Fig. 3). The marked downregulation of D1, whose activity is unrelated to the nuclear fraction of the TH receptor-bound T3, paralleled by enhanced D2 expression, supports the concept that D2 re-expression in ATC does not likely reflect the primary thyroid function to release T3, but rather the requirement to increase the intracellular nuclear T3 concentration in ATC cells. This concept is supported by data on ATC cells obtained upon D2 depletion, which indicate a requirement of D2 action in these cells to survive and maintain their cancer phenotypes (De Stefano *et al.* accompanying manuscript). The second clue on the potential role of D2 in ATC tumorigenesis came from an ATAC-seq analysis performed in ATC from mice carrying *BRAF^V600E^* mutation and *SWI/SNF* loss (Saqcena *et al.* 2021). This model indicated that the loss of three different subunits of SWI/SNF chromatin remodeling complexes impairs the expression of thyroid differentiation genes, thereby promoting the progression of PTC to an undifferentiated state. In particular, the loss of the SMARCB1 subunit leads to anaplastic transformation in about 90% of *BRAF^V600E^*-mutated PTCs. Strikingly, the *BRAF^V600E^/SMARCB1*-mutated ATC showed similar levels of chromatin accessibility at *DIO2* locus than wildtype controls, but impaired accessibility at *DIO1* consistent with the loss of expression of other thyroid differentiation genes, with the exception of* DIO2*.

In line with these results, in a recent study from our group (De Stefano *et al.* accompanying manuscript), we found that *DIO2* is highly expressed in two ATC cell lines (i.e. 8505c and KMH2) compared to PTC cells lines (i.e. K1 and TPC-1), while *DIO1* is strongly downregulated in both cell models compared to normal thyroid cells. We also found that ATC cells upregulate the expression of thyroid hormone nuclear receptor (THR) α while suppress the THRβ (which is the major isoform expressed in normal thyroid), possibly suggesting that the D2-derived T3 in ATC acts on a different subset of target genes through the expression of a different THR isoform. Most importantly, our study showed that D2-blocking in ATC induced cell senescence and significantly blunted their invasive potential.

Overall, D3 upregulation in PTC suggests that low intracellular T3 level is paralleled by a low proliferation rate and relatively slow tumor growth, while the D2 upregulation in ATC (i.e. higher intracellular T3) is needed in rapidly growing tumors. However, in PTC cells, we observed that an enhanced intracellular T3 is accompanied by senescence (unpublished data) suggesting that it is not the absolute intracellular T3 concentration that promotes/hampers tumor proliferation, rather its adjustment is cell-context dependent and represents a cell-specific requirement in thyroid cancer cells. The pathological meaning of the higher intracellular T3 requirements in ATC compared to PTC still need to be investigated. Among multiple mechanisms, the increase in D2-derived T3 in ATC may support the increased energy consumption consequent to its highly proliferative state. In such a situation, cancer cells enhance aerobic glycolysis as a source of ATP for the increased energy request (Bao *et al.* 2021). Notably, T3 increases the expression of glycolytic genes (such as aldolase A, enolase, pyruvate kinase M2, and lactate dehydrogenase A) (Miro *et al.* 2021), as well as the glucose transporter genes GLUT1, 3, and 4 (Casla *et al.* 1990).

The hypothesis that D2 is associated with cancer dedifferentiation in thyroid tumorigenesis has to be confirmed, but it would be in line with observations in other cancer contexts. In skin squamous cell carcinoma (Miro *et al.* 2019) and colon cancer (Dentice *et al.* 2012), we showed that D3 is dramatically downregulated at later phases of tumorigenesis and that the expression of D2 is upregulated in high-grade squamous cell carcinoma. High D2 expression levels have also been found in other advanced cancer cells vs their respective normal cells, such as rhabdomyosarcoma (RMS-13) (da-Silva *et al.* 2007) and mesothelioma cell lines (MSTO-211H) (Curcio *et al.* 2001). The reciprocal opposite changes in D2 and D3 expression increase the intracellular concentration of T3, but the role of the T3 signaling in cancer progression is largely unknown. The identification of the ZEB-1 pathway as positively induced by T3 seems to indicate that T3 is required for the fully advanced and metastatic properties of cancer cells (Miro *et al.* 2019).

**Figure 4:**
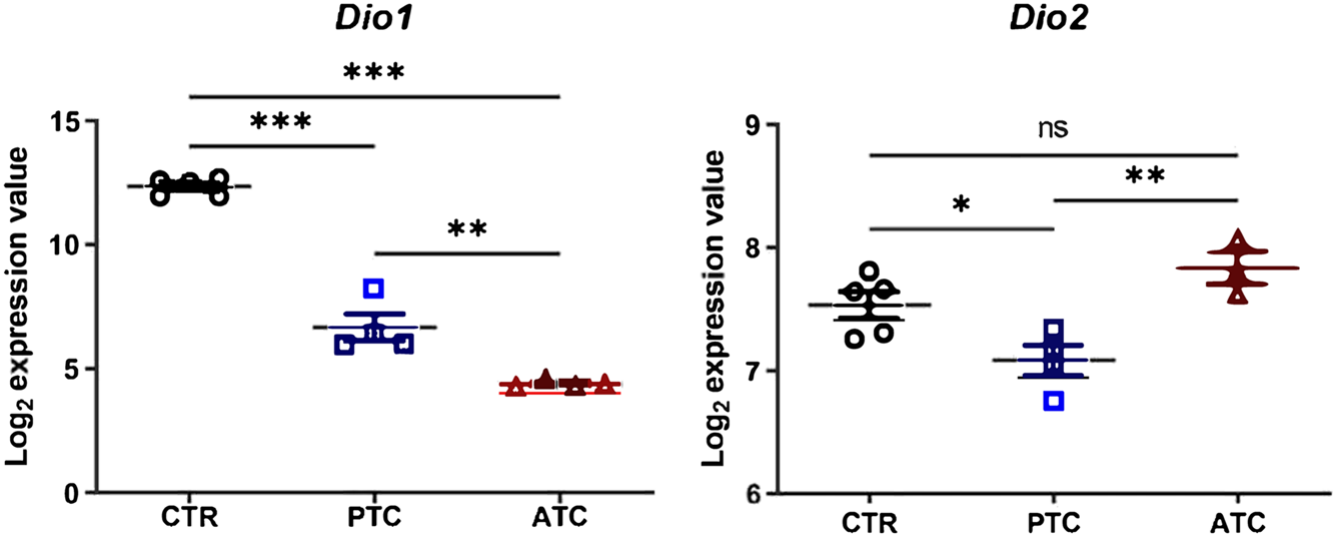
Array analysis of type 1 deiodinase gene (*Dio1*) and type 2 deiodinase gene (*Dio2*) expression in a mouse model of *BRAF^V600E^*-mutated PTC and *BRAF^V600E^/TP53* ATC (from (Knauf *et al.* 2018). Dataset from GSE118022 was analyzed using GEO2R. *P < 0.05; **P < 0.01; ***P < 0.001.

What are the molecular drivers of D2 in ATCs? We do not know the answer yet, neither whether D2 upregulation only occurs in the epithelial ATC cells or also in the stromal/immune cell components, which are particularly relevant in the bulk of ATC (Landa *et al.* 2016). However, in our aforementioned study (De Stefano *et al.* accompanying manuscript), we found that a mutant p53 transfected in PTC cells potently induces *DIO2* expression. Given that mutated p53 is a major driver of PTC to ATC transition, this observation helps to understand the expression of D2 in ATC with mutated p53. Supporting this hypothesis, we conducted an *in silico* analysis of deiodinase expression of murine *BRAF^V600E^*-mutated PTC and *BRAF^V600E^*/*TP53* ATC from arrays derived from a recent study of Knauf *et al.* (Knauf *et al.* 2018) (Fig. 4). Strikingly, we found that D2 expression is upregulated in ATC compared with PTC, wherein it is even higher than in normal thyroid tissue. Conversely, D1 expression in ATC is similarly absent as in PTC, that is, significantly lower than in normal thyroid tissue. These results are consistent with the expression data in human ATC and prompt the need for further investigations of the potential role of D2 in ATC tumorigenesis.

## Conclusions

The presence of a specific modulation in the expression of deiodinases during thyroid tumorigenesis suggests that not only changes in TH metabolism occur early in thyroid tumorigenesis but also that initial and late-phase thyroid cancers require different intracellular TH concentrations. Strictly depending on tumor stage, the TH signaling seems to be able to either impair (in differentiated cancer) or promote (in undifferentiated) the tumor growth. The opposite variations in intracellular TH signaling between low- and high-grade thyroid cancers predicted from these divergent deiodinase expressions shed new light on the role of TH in tumor progression. Far from being only related to lack of thyroid differentiation, the significant D2 expression in ATC suggests that the elevated TH signaling is active in high-grade thyroid cancers. This represents a novel and neglected aspect of thyroid tumorigenesis that raises important questions on the role of TH action in tumor progression and requires deeper studies. The accumulating knowledge in TH signaling in thyroid cancer will possibly allow to identify selected actors of TH signaling as new potential stage-specific target in thyroid cancer treatment.

## Declaration of interest

All authors declare no competing interests.

## Funding

This study was supported by AIRC Individual Grant to DS (IG 2022, Project Number 27729) and by National Center for Gene Therapy and Drugs based on RNA Technology MUR-CN3 CUP E63C22000940007 to DS.

## Author contribution statement

All authors contributed to the manuscript and approved its final version.
